# Knowledge, practices, and barriers to early mobilization of critical care nurses in three hospitals in Sabah, Malaysia: A multicentre cross-sectional study

**DOI:** 10.1016/j.mex.2026.103901

**Published:** 2026-04-03

**Authors:** Saniah Ajui, Boon Tat Yeap, Deena Clare Thomas

**Affiliations:** a Department of Nursing, Faculty of Medicine and Health Science, Universiti Malaysia Sabah, Malaysia; b Cardiothoracic Department, Hospital Queen Elizabeth II, Kota Kinabalu, Sabah, Malaysia; c Department of Anaesthesiology and Intensive Care, Faculty of Medicine and Health Science, Universiti Malaysia Sabah, Malaysia

**Keywords:** Early mobilisation, Intensive care unit, Nursing practice, Survey, Clinical, Questionnaire

## Abstract

Early mobilization (EM) is increasingly recognized as an important intervention in intensive care units (ICUs) to reduce complications associated with prolonged immobilization, including ICU-acquired weakness, delayed recovery, and longer hospital stay. Despite strong evidence supporting its benefits, EM implementation remains inconsistent in practice and may be influenced by variations in nurses’ knowledge, clinical practices, and perceived barriers, particularly in resource-constrained settings. In Sabah, Malaysia, limited empirical evidence exists to systematically assess these factors across ICU settings. This paper presents a reproducible cross-sectional protocol to evaluate ICU nurses’ knowledge, practices, and perceived barriers toward early mobilization across multiple centres in Sabah. The study uses proportionate stratified sampling and a structured self-administered questionnaire adapted from validated instruments, covering sociodemographic characteristics, knowledge, practice, and perceived barriers. Knowledge will be assessed using a standardized scoring system based on Bloom’s cut-off classification and reported as percentage scores categorized into low, moderate, and high levels. Practice patterns and perceived barriers will be summarized descriptively, including the most frequently reported barriers. This protocol offers a practical and scalable approach to assess EM implementation in ICU settings and may help identify gaps that inform targeted training, protocol refinement, and future implementation strategies.

## Graphical abstract

**Figure:**
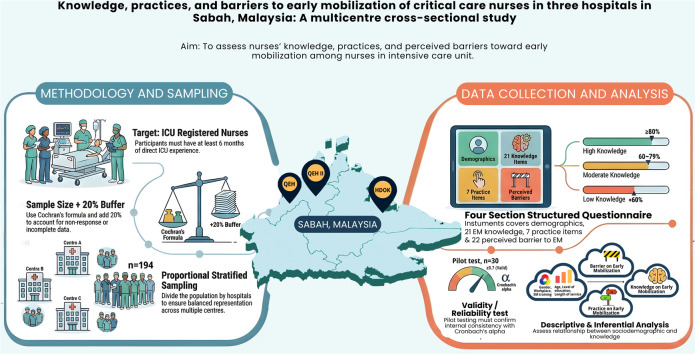

## Specifications table

**Table:** 

**Subject area**	Medicine and Dentistry
**More specific subject area**	early mobilization; critical care nursing; nursing research
**Name of your protocol**	Knowledge, practices, and barriers to early mobilization of critical care nurses: A multicenter cross-sectional study in Sabah, Malaysia
**Trial registration**	OSF Registration DOI: https://doi.org/10.17605/OSF.IO/JFX42
**Ethics**	Ethical approval was obtained from the Medical Research and Ethics Committee (MREC), Malaysia via the National Medical Research Register (NMRR) (Registration No.: 25–03,901-4CL) for the study titled “Nurses’ Perceived Barriers to Early Mobilization in Intensive Care Unit: A Multi-Center Cross-Sectional Study.” Participation in the study was voluntary, and consent was obtained electronically before respondents accessed the questionnaire. No personal identifiers were recorded, and all data were collected anonymously and handled with strict confidentiality. Participants were also informed that they could discontinue their participation at any point without any implications.
**Value of Protocol**	This protocol is valuable for: • providing a structured approach to assess nurses’ knowledge, practices, and perceived barriers related to early mobilization in ICU settings; • generating context-specific evidence on early mobilization practices where local data are limited; • identifying key factors influencing implementation, which can inform targeted training, protocol development, and resource planning; • supporting healthcare institutions in improving consistency and quality of early mobilization practices to enhance patient outcomes.

## Background

Early mobilization (EM) has become an integral component of contemporary intensive care practice due to its role in reducing complications associated with prolonged immobilization. Critically ill patients are highly susceptible to adverse outcomes such as muscle wasting, ICU-acquired weakness, delirium, and prolonged dependence on mechanical ventilation when mobility is delayed [[1], [2], [3]]. Evidence suggests that the loss of muscle mass can occur rapidly during critical illness, contributing to long-term functional impairment and reduced quality of life after discharge [[4], [5], [6]].

To mitigate these risks, EM has been widely recommended as a safe and feasible intervention when initiated in clinically stable patients. Evidence from prior research indicates that early mobilization improves patients’ functional status, decreases the need for prolonged mechanical ventilation, shortens ICU length of stay, and supports better recovery outcomes [[7], [8], [9], [10]]. In addition, early mobilization has been associated with psychological benefits, including reduced delirium and improved patient engagement during ICU stay [11,12].

Despite strong evidence supporting its effectiveness, the implementation of EM remains inconsistent across clinical settings. The translation of evidence into practice is often influenced by multiple factors, including healthcare providers’ knowledge, attitudes, and confidence, as well as organizational readiness and resource availability [[13], [14], [15]]. Nurses, as primary caregivers in ICU settings, play a central role in initiating and sustaining mobilization activities, making their competencies and perceptions critical to successful implementation [16,17].

However, several barriers continue to limit the integration of EM into routine practice. These barriers are multifactorial and may include patient-related factors such as clinical instability and sedation, nurse-related factors such as lack of training or fear of adverse events, and system-level challenges including inadequate staffing, absence of standardized protocols, and limited equipment [[18], [19], [20], [21]] . These challenges contribute to variations in practice and may delay or prevent the delivery of optimal care.

In Malaysia, national guidelines have been established to promote EM in ICU settings [10]. In addition, evidence from Sarawak has reported early mobilization practices among ICU clinicians, providing relevant local insight into current implementation patterns [22]. However, adherence to EM remains suboptimal, with mobilization practices often limited to passive interventions [23,24]. Furthermore, existing studies are largely concentrated in selected regions, with limited data available from Sabah. Differences in healthcare infrastructure, workforce distribution, and resource availability may further influence the implementation of EM in this context. Similar challenges have been reported internationally, including in recent studies from Palestine and Southern West Bank hospitals, where EM implementation was influenced by nurse-related, patient-related, and institutional constraints. These findings suggest that barriers to early mobilization may be shared across resource-constrained settings, although their relative importance may vary according to local infrastructure and workforce contexts [25,26].

Given these gaps, there is a need for a structured approach to examine nurses’ knowledge, practices, and perceived barriers related to EM within the local setting. Understanding these factors is essential to support evidence-based improvements and enhance the consistency of EM implementation in ICU care. Therefore, this study aims to: (1) assess ICU nurses' knowledge of early mobilization; (2) describe current EM practices; (3) identify perceived barriers to EM implementation; and (4) examine the relationship between sociodemographic characteristics and knowledge levels.

## Description of protocol

### Review question

Primary objective

To assess nurses’ knowledge, practices, and perceived barriers toward early mobilization among nurses in intensive care unit.

Secondary objectives

This study also aims to:

• determine the level of knowledge among nurses regarding early mobilization;
• describe current practices related to early mobilization in ICU settings;
• examine differences in mobilization practices across multiple centers;
• explore the relationship between nurses’ sociodemographic characteristics and their knowledge of early mobilization.
• To identify the perceived barriers to early mobilization of patients in the ICU.

### Study design

A multi-centre, cross-sectional observational study will be conducted to assess nurses’ knowledge, practices, and perceived barriers related to early mobilization in ICU settings. Data will be collected at a single time point using a structured questionnaire.

### Study sample

The study population comprises registered nurses working in ICUs across three public hospitals in Sabah, Malaysia: Hospital Queen Elizabeth (QEH), Hospital Queen Elizabeth II (QEH II), and Hospital Duchess of Kent (HDOK). These centres represent different ICU service contexts, including general ICU, cardiothoracic ICU, mixed medical-surgical ICU and mixed medical-surgical neuro ICU with approximate bed capacities of 42 beds (QEH I), 16 beds (QEH II), and 18 beds (HDOK), respectively. Based on staffing records, the ICU nurse population across the three centres is 278, comprising 165 nurses from QEH, 57 from QEH II, and 56 from HDOK. A proportionate stratified sampling approach will be used to ensure representation from each centre.

### Inclusion/ exclusion criteria

**Inclusion criteria**

• Registered nurses currently working in ICU;
• Minimum of six months ICU experience;
• (6) Direct involvement in early mobilization activities based on self-reported by nurses;
• Willingness to participate with informed consent.

**Exclusion criteria**

• Nurses on maternity or study leave during data collection;
• Nurses not involved in mobilization practices;
• ICU experience less than six months;
• Refusal to participate.

**Participant’s’ recruitment**

• A proportionate stratified sampling approach will be used, with each hospital serving as a stratum. Within each hospital, eligible nurses will be randomly selected in proportion to the size of the ICU nursing workforce.
• Nurses who meet the inclusion criteria will be randomly selected and approached via email. A survey link will be distributed to the selected participants.
• Participation will be voluntary, and informed consent will be obtained electronically prior to accessing the questionnaire.
• Direct involvement in early mobilization activities will be determined based on self-report by nurses who indicate that mobilization forms part of their routine ICU clinical responsibilities.

**Data collection and management**

• Data collection is expected to take place over approximately two months, or until the required sample size is achieved. Regular reminders will be issued to improve response rates.
• The questionnaire is adapted from previously validated instruments, and responses will be recorded anonymously without collection of identifiable information. Data will be securely stored and accessible only to the research team.
• Data will be collected using a self-administered online questionnaire consisting of four sections (refer Table 1): Section A- Sociodemographic characteristics; Section B- Knowledge on Early Mobilization; Section C- Practice on Early Mobilization; and Section D- Perceived Barriers related to Early Mobilization.

**Table 1:** Summary of research instruments and analytical approach.

Section	Construct	Description	Measurement & Scoring	Interpretation	Analytical Approach
A	**Sociodemographic Characteristics**	5 items capturing participants’ background information including age, gender, education level, workplace, and length of ICU experience	Categorical and ordinal variables (coded numerically for analysis)	Used as independent variables to describe sample characteristics and examine associations with level of knowledge	- Frequency & percentage - Descriptive summary - Used in inferential analysis (e.g., chi-square, correlation)
B	**Knowledge on Early Mobilization**	21-item questionnaire assessing knowledge on EM concepts, types, initiation criteria, monitoring, and discontinuation	Correct = 1; Incorrect/Unsure = 0. Total score: 0–21. Converted to percentage	Continuous: Higher score = better knowledge Categorical: Low (<60 %), Moderate (60–79 %), High (80–100 %)	- Mean, SD, score distribution - Frequency & % (knowledge levels) - Inferential tests (e.g., correlation, chi-square)
C	**Practice of Early Mobilization**	7-item multiple-response questionnaire assessing types of mobilization, frequency, and adherence to clinical criteria	Categorical/multiple response items; no composite score; descriptive analysis only.	Interpreted at three levels: Type: activities performed Frequency: consistency of practice Appropriateness: adherence to clinical criteria	- Frequency & percentage - Pattern identification (type & frequency) - Comparison across centres
D	**Perceived Barriers to Early Mobilization**	22-item questionnaire assessing patient-, nurse-, and system-related barriers	Agree = 1; Disagree = 0. Total score: 0–22	Total score: Higher score = more perceived barriers Item level: identifies most common barriers	- Frequency & % per item - Ranking of barriers - Score distribution
• Details of each section are as follows:

**Section A: Sociodemographic Characteristics**

This section gathers background information about the participants to support subgroup analysis and examination of associations with outcome variables.

It includes five items:

• age
• gender
• workplace (hospital)
• level of education
• length of service in ICU

All variables are numerical / categorical / ordinal in nature and are used as independent variables in subsequent analyses.

**Section B: Knowledge on Early Mobilization**

**Description**

Knowledge was assessed using a structured questionnaire adapted from a previously validated instrument [9], designed to evaluate nurses’ understanding of early mobilization principles and relevant clinical considerations.

The instrument comprises **21 items** covering the following domains:

• definition and concept of early mobilization
• scope and types of mobilization activities
• criteria for initiation
• monitoring indicators during mobilization
• criteria for discontinuation

Each item is presented with three response options:

• “Yes”
• “No”
• “Unsure”

**Scoring and Interpretation**

1. Total Knowledge Score (Continuous variable)

Each item is scored as follows:

• Correct response = 1
• Incorrect response = 0
• “Unsure” response = 0 (Unsure” responses are scored as incorrect because they indicate absence of correct operational knowledge at the point of decision-making, which is functionally similar to an incorrect response in clinical practice).

The total knowledge score is calculated by summing all item responses, with a possible range of 0 to 21. The score is further converted into a percentage value to improve interpretability.

• Higher scores indicate better knowledge of early mobilization
• Lower scores indicate limited understanding

This continuous score will be used for statistical analysis.

2. Knowledge Level Classification (Categorical variable)

For interpretation purposes, total knowledge scores will be converted into percentage scores and categorized based on Bloom’s cut-off points as follows:

• Low knowledge (<60 %)
• Moderate knowledge (60 %–79 %)
• High knowledge (≥80 %).

These percentage thresholds correspond to raw score ranges of 0–12, 13–16, and 17–21, respectively. This categorization allows meaningful grouping of respondents and supports comparison across different subgroups.

**Analytical Approach**

Knowledge will be analysed at two complementary levels:

1. Continuous level (score-based analysis):
○ mean, standard deviation, and score distribution
○ used for inferential analysis (e.g., correlation)
2. Categorical level (classification-based analysis):
○ frequency and percentage of knowledge levels (low, moderate, high)
○ used for descriptive interpretation and group comparison

This dual approach enables both detailed statistical analysis and practical interpretation of knowledge levels among ICU nurses.

**Section C: Practice of Early Mobilization**

**Description**

Practice was assessed using a questionnaire adapted from previous research [15], designed to capture nurses’ actual implementation of early mobilization in ICU settings.

This section consists of seven multiple-response items describing:

• types of mobilization activities performed (e.g., passive exercise, sitting, standing, ambulation)
• frequency of mobilization practices
• adherence to clinical criteria for safe mobilization

The practice items are framed based on established clinical parameters, including:

• hemodynamic stability (e.g., systolic blood pressure >90 mmHg, heart rate <120 bpm)
• absence of acute cardiac instability or dysrhythmia
• adequate oxygenation (e.g., FiO₂ ≤ 0.6, PEEP ≤ 10 cmH₂O, SpO₂ >90 %)

**Interpretation of Practice Data**

Unlike knowledge and perceived barriers, practice is not measured using a composite score. Instead, it is interpreted descriptively at multiple levels to reflect real-world clinical behavior.

1. Type of Practice (Activity Level)

Practice is interpreted based on the proportion of nurses performing specific mobilization activities. This provides insight into the extent and progression of mobilization practices, from basic (e.g., passive exercise) to advanced (e.g., ambulation).

2. Frequency of Practice

The frequency of reported activities is analysed to determine how consistently early mobilization is implemented in clinical settings. This helps identify whether mobilization is performed routinely or only occasionally.

3. Clinical Appropriateness

The practice section includes items that ask whether nurses consider specific physiological criteria before initiating mobilization, including hemodynamic stability, cardiac rhythm stability, and oxygenation adequacy. Clinical appropriateness will therefore be interpreted based on respondents’ reported consideration of these predefined safety criteria rather than by deriving a separate composite appropriateness score. This reflects adherence to safe practice standards.

**Analytical Approach**

Practice data will be analysed using:

• frequency and percentage distributions
• categorization of mobilization activities by type and frequency

This approach enables identification of:

• dominant patterns of practice
• variation across centres
• gaps between recommended and actual practice

**Section D: Perceived Barriers to Early Mobilization**

**Description**

Perceived barriers were assessed using a structured instrument adapted from a previously validated questionnaire [12], designed to identify factors that may hinder the implementation of early mobilization in ICU settings.

This section comprises 22 items, representing three domains of barriers:

• patient-related factors (e.g., hemodynamic instability, sedation, presence of invasive devices)
• nurse-related factors (e.g., lack of knowledge, fear of causing harm, workload)
• system-related factors (e.g., staffing limitations, lack of equipment, absence of standardized protocols)

Each item is measured using a binary response format:

• Agree = 1 (barrier present)
• Disagree = 0 (barrier not present)

**Scoring and Interpretation**

1. Total Barrier Score (Overall Level)

A total barrier score is calculated by summing responses across all 22 items, with a possible range of 0 to 22.

• Higher scores indicate that respondents perceive a greater number of barriers to implementing early mobilization.
• Lower scores indicate fewer perceived barriers.

This total score reflects the overall burden of perceived barriers and will be used for:

• descriptive analysis (e.g., mean and distribution)

2. Item-Level Analysis (Specific Barrier Identification)

In addition to the total score, each barrier item will be analysed individually using:

• frequency and percentage of agreement

This allows identification of:

• the most commonly reported barriers
• patterns across different domains (patient, nurse, system)

Items will be ranked based on the proportion of respondents who agree with each statement, enabling prioritization of key challenges in clinical practice.

**Analytical Approach**

Perceived barriers will therefore be interpreted at two levels:

1. Overall level (total score) - to determine the general extent of perceived barriers among nurses
2. Item level (individual barriers) - to identify specific factors that most frequently hinder early mobilization

This dual approach ensures both quantitative assessment of barrier burden and practical identification of priority areas for intervention. For interpretive purposes, barriers endorsed by >50 % of respondents will be considered major barriers, as these represent the most commonly perceived obstacles to early mobilization and may warrant prioritization in future interventions.

**Validity and Reliability**

The instruments used in this study were adapted from previously validated questionnaires. Reported internal consistency for the original tools ranged from Cronbach’s alpha 0.73 to 0.78 [9,12,15]. A pilot study involving approximately 30 ICU nurses will be conducted to:

• assess clarity and feasibility
• evaluate internal consistency
• ensure suitability within the local context

If any scale demonstrates a Cronbach’s alpha below 0.70 during pilot testing, this will be acknowledged as a limitation in terms of internal consistency within the local context, and the affected items will be reviewed for clarity and contextual relevance before final data collection.

**Sample Size**

The sample size was calculated using Cochran’s formula with adjustment for a finite population, based on the total number of ICU nurses (N = 278) across the three participating hospitals. Using a 95 % confidence level and a 5 % margin of error, the initial sample size was determined to be 162 participants. To compensate for possible non-response and incomplete questionnaires, an additional 20 % was added, resulting in a final target sample of 194 nurses. A proportionate stratified sampling approach was applied to ensure adequate representation from each hospital, with sample allocation based on the size of the nursing workforce in each centre.

**Potential Risks**

Participation in this study involves minimal risk, as data are collected using a self-administered questionnaire. No clinical intervention or direct patient involvement is required.

However, minor concerns may arise from:

• perceived discomfort when responding to questions related to workplace practices;
• concerns about professional judgment or evaluation.

To address these, the questionnaire is anonymous, and participants are informed that responses will be used solely for research purposes.

**Risk of Bias**

Several potential sources of bias are acknowledged:

• Response bias: Participants may provide socially desirable responses, particularly regarding their practices or knowledge.
• Self-report bias: Data are based on self-reported measures, which may not fully reflect actual clinical practice.
• Selection bias: Participation is voluntary, and those with greater interest in early mobilization may be more likely to respond.
• Recall bias: Participants may have difficulty accurately recalling the frequency or type of mobilization activities performed.

Efforts to minimize bias include anonymous data collection, clear questionnaire instructions, and the use of previously validated instruments.

### Statistical analysis

Data analysis will be performed using IBM SPSS Statistics version 29. Both descriptive and inferential statistical methods will be applied. Descriptive analysis will involve:

• frequencies and percentages for categorical variables;
• means and standard deviations for continuous variables.

Inferential analysis will include:

• one-way ANOVA or Kruskal–Wallis test to compare practice across hospitals, depending on data distribution;
• chi-square test or Fisher’s exact test to examine associations between categorical variables;
• Pearson correlation for relationships between continuous variables, where assumptions are met.

A significance level of p < 0.05 will be used, and appropriate assumption checks will be performed prior to analysis.

### Ethical aspects

Ethical clearance for this study was granted by the Medical Research and Ethics Committee (MREC), Malaysia, via the National Medical Research Register (NMRR) (Registration No.: 25–03,901-4CL) for the study titled *“Nurses’ Perceived Barriers to Early Mobilization in Intensive Care Unit: A Multi-Center Cross-Sectional Study.”*

Participation was voluntary, and informed consent was obtained electronically prior to data collection. Participants were provided with clear information regarding the study purpose and procedures, and were free to withdraw at any stage without any consequences.

### Data confidentiality and privacy

All data collected in this study are anonymous and non-identifiable. No personal identifiers such as names or staff identification numbers are recorded. Data will be used solely for research purposes and reported in aggregate form. Access to the dataset is restricted to the research team, and all electronic data will be stored securely.

### Protocol validation

This protocol provides a structured approach to examine nurses’ knowledge, practices, and perceived barriers related to early mobilization in ICU settings within Sabah, where limited empirical evidence is currently available. The study addresses an important gap by generating context-specific data that reflect real clinical practices and challenges in the local setting. The questionnaire used in this study is adapted from previously validated instruments, ensuring methodological robustness while allowing contextual relevance. Content validity is established through expert review by critical care nursing specialists, and reliability is assessed through a pilot study using internal consistency measures.

The combination of validated tools and local adaptation strengthens the applicability and credibility of the protocol.

The findings from this study are expected to support evidence-based improvements in ICU practice, particularly in enhancing early mobilization implementation through targeted training, protocol refinement, and resource planning.

### Limitations

This study has several limitations. First, the cross-sectional design restricts the ability to establish causal relationships, as data are collected at a single point in time. Second, the study relies on self-administered questionnaires, which may introduce response bias, including social desirability and recall bias. In addition, direct involvement in early mobilization is determined by self-report, which may result in some misclassification and introduce further bias. Although proportionate stratified sampling is used, the study is limited to three hospitals in Sabah, Malaysia, which may affect the generalizability of the findings to other regions, institutions, or healthcare settings. Furthermore, the exclusion of nurses with less than six months of ICU experience may limit the applicability of the findings to novice ICU nurses.

The measurement approach also has some limitations. The binary response format used for perceived barriers can identify whether a barrier is present, but it does not capture the severity, relative importance, or intensity of that barrier. Variations in participants’ interpretation of questionnaire items may also influence responses, despite efforts to enhance clarity through pilot testing. In addition, if pilot testing yields a Cronbach’s alpha below 0.70 for any scale, this may indicate lower internal consistency of the instrument within the local context and should be taken into account when interpreting the findings. Finally, although efforts are made to minimise bias, unmeasured confounding factors and other inherent limitations of observational research may still influence the results.

## Supplementary material and/or additional information

Supplementary material 1 includes the self-administer questionnaire to be use for data collection: Section A – Section D.

## Data availability

The research protocol for this study has been preregistered on the Open Science Framework (OSF) and can be accessed at: https://doi.org/10.17605/OSF.IO/JFX42.

## Declaration of the use of artificial intelligence and large language models (LLMs)

Selected portions of this manuscript, particularly those related to narrative synthesis and formatting, were developed with assistance from an AI-based language tool (ChatGPT, GPT-5, OpenAI), under the guidance and oversight of the authors. The tool was utilized solely to improve language expression, structural coherence, and clarity of presentation. All interpretation of findings, critical evaluation, and final approval of the content were conducted exclusively by the authors. The authors assume full responsibility for the accuracy, integrity, and originality of the work.

## Related research article

None.

## Declaration of competing interest

The authors report no conflicts of interest related to this study.

## Appendix Supplementary materials

Image, application 1
