# Supplementary material for: Knowledge, practices, and barriers to early mobilization of critical care nurses in three hospitals in Sabah, Malaysia: A multicentre cross-sectional study
Source: MethodsX. 2026 Apr 3;16:103901. doi: 10.1016/j.mex.2026.103901 (PMC13090670; doi:10.1016/j.mex.2026.103901)
Supplement: Supplementary file 1 [file mmc1.pdf]

QUESTIONNAIRE

TITLE: Knowledge, practices, and barriers to early mobilization of critical care nurses

Instruction: This questionnaire consists of 4 parts. Section A, Section B, Section C and Section D. Kindly complete the questionnaire by placing or filling (/) in the space provided.

Section A : Sociodemographic Data

Age (years)	:	______________________			
Gender	:	☐	Male	☐	Female
Educational level	:	☐	Diploma	☐	Advanced Diploma
		☐	Degree	☐	Advanced Diploma & Degree
Length of service (years)	:	______________________			
Workplace	:	☐	ICU 1 (QEH)	☐	ICU 2 (QEH)
		☐	ICU 1 (QEH II)	☐	CICU (QEH II)
		☐	ICU HDOK		

Pre-requisite knowledge related to early mobilization

Ever attend training programs regarding early mobilization of critically ill patients?	Yes	☐
	No	☐
Have you ever been exposed to or read any protocol or guideline on early mobilization in the ICU?	Yes	☐
	No	☐

QUESTIONNAIRE

TITLE: Knowledge, practices, and barriers to early mobilization of critical care nurses

Section B : Knowledge of early mobilization in the ICU

No	Item	Yes	No
1	Early mobilization refers to physical therapy administered to patients by medical staff within one week of ICU admission		
2	Early mobilization is an element of the ABCDE bundle (awakening, breathing, coordination, delirium monitoring, early mobilization)		
3	Early mobilization only includes active and passive exercise in bed		
4	Assisting patients with exercise could help promote alertness, increase perfusion, and trigger spontaneous breathing		
5	Early mobilization could improve patients' respiratory function		
6	Patients on mechanical ventilation should be strictly in bed and are forbidden to move to the ground to prevent danger and injury		
7	Bedside cycling could be used for active and passive activity		
8	Electrical muscle stimulation is a kind of early mobilization for ICU patients		
9	Early mobilization is unrelated to the incidence and severity of delirium in ICU patients		
10	The frequency of early mobilization for ICU patients is one to two times per day		
11	The appropriate duration of early mobilization is generally 30 minutes each time		
12	Early mobilizations should be suspended if systolic blood pressure is over 160 mmHg		
13	Patients who use vasoactive drugs to maintain hemodynamic stability cannot perform active exercise (i.e. bedside sitting and ambulation activities.)		
14	Early mobilization should consider the patient's condition, follow the principle of gradual progress, begin with passive activity, and gradually transition to active training		
15	Patients' early mobilization plans include type, frequency, time, and intensity		
16	Patients' early mobilization should consider patients' sedation and analgesia status.		
17	RASS score should be used as an assessment indicator for early mobilization, and early mobility can be performed only if the RASS score is more than 1		
18	ICU-AW occurrence could increase patients' mortality risk and impact patients' rehabilitation		
19	Long-term bed confinement and immobility is one of the significant risk factors for developing ICU-AW		
20	MRC sum scores could be used to diagnose ICU-AW		
21	Early mobilization performance could decrease ICU-AW incidence and improve patients' clinical outcomes		

QUESTIONNAIRE

TITLE: Knowledge, practices, and barriers to early mobilization of critical care nurses

Section C : Practice on Early Mobilization

The criteria for patient in this section was :

i. Myocardial stability with systolic blood pressure >90 mmHg, heart rate <120 beats/min, no evidence of acute myocardial ischaemia in the last 24 hr and absence of dysrhythmia requiring new anti-dysrhythmic agents in the last 24 hr.

ii. Oxygenation adequacy (O) was defined as patients who met the parameters of $FiO_2 \leq 0.6$, PEEP $\leq$ 10 cm H_2O, $SPO_2 > 90\%$ and respiratory rate < 35 per minute.

No	Practice	Invasive Ventilated Patient		Non-invasive & non-ventilated patient	
		Yes	No	Yes	No
1	Passive range of movement				
2	Two Hourly Turning				
3	Sitting in Bed				
4	Sitting on the edge of the bed				
5	Sitting out of bed (armchair)				
6	Walking on the spot				
7	Walking				

No	Frequency of performing EM per Shift	0	1	2
1	**Invasive Ventilated Patient**			
	AM SHIFT			
	PM SHIFT			
	NIGHT SHIFT			
2	**Non-invasive & non-ventilated patient**			
	AM SHIFT			
	PM SHIFT			
	NIGHT SHIFT			

QUESTIONNAIRE

TITLE: Knowledge, practices, and barriers to early mobilization of critical care nurses

Section D: Barrier on early mobilization

No	Item on barrier	Agree	Disagree
1	Hemodynamic instability of patient		
2	Sedated patient		
3	Dislodgement of connected devices		
4	Obese patient		
5	High severity of pain		
6	Poly trauma		
7	Raise the patient’s temperature		
8	Patient with agitation or delirium		
9	Presence IV connections or drains		
10	Respiratory instability/dyspnea		
11	The patient's belief that bed rest and immobility equals recovery		
12	Lack of self-directed learning		
13	Inadequate training regarding early mobilization		
14	Lack of nursing skills		
15	In appropriate nurse patient ratio		
16	Early mobilization is not a priority		
17	Fear of harm/ injury to patient		
18	Excess work		
19	Not enough time		
20	No early mobilization protocol		
21	Lack of special mobility chair		
22	Need for doctor’s order		

Thank you for your time. You have greatly contributed to this research.
